# Pennies for Your Thoughts: A Case Series of Pancytopenia Due to Zinc-induced Copper Deficiency in the Same Patient

**DOI:** 10.5811/cpcem.2019.7.43697

**Published:** 2019-10-14

**Authors:** Bram A. Dolcourt, James H. Paxton, Keenan M. Bora, Cynthia K. Aaron

**Affiliations:** Wayne State University, Department of Emergency Medicine, Detroit, Michigan

## Abstract

A 47-year-old schizophrenic male presented on three separate occasions with pancytopenia and sideroblastic anemia due to copper deficiency from massive zinc penny ingestion. The poisoning was treated differently on each visit: intravenous (IV) copper plus surgical decontamination and chelation with calcium disodium versenate (CaNa2EDTA); IV copper plus whole bowel irrigation; and IV copper with surgical decontamination only. Serum zinc half-lives were 80.0 hours, 233.2 hours, and 83.9 hours, respectively. Importantly, chelation with CaNa2EDTA did not significantly alter the elimination half-life. This is the first reported case of the same patient being treated on three different occasions with three different regimens for this condition.

## INTRODUCTION

Copper deficiency due to zinc toxicity from ingested pennies is a rare entity. Since 1982, all United States one-cent coins have been minted from zinc with copper-plating.^1^ Zinc-containing pennies are ubiquitous in this country, and their small size makes them easily ingestible. Treatment of zinc toxicity typically includes a combination of gastric decontamination and copper supplementation, with or without calcium disodium versenate (CaNa_2_EDTA) chelation.^2^–^10^ The existing literature on this subject is currently limited to individual case reports and speculation about ideal treatment. Unfortunately, little comparative data exists to help determine which treatment may be superior for eliminating zinc or even improving clinical outcomes.

Since the elimination kinetics of zinc are not well understood, enhanced elimination practices have not yet been established. It is not known whether chelation confers significant benefit, as there is limited data in humans.^11^ We present the case of an individual with recurrent severe zinc poisoning presenting to our institution on three separate occasions, and treated with three different treatment regimens based on variations in his clinical presentation. This natural experiment provides data on zinc elimination associated with different treatment modalities. Pawa et al. published the first presentation and hospital course for this patient in 2008.^9^

## CASE REPORT

A 47-year-old male patient with a history of schizophrenia and human immunodeficiency virus presented on three separate occasions with pancytopenia due to copper deficiency resulting from severe zinc toxicity. He was a habitual consumer of pennies, as he felt it improved his singing voice. His first emergency department (ED) visit was precipitated by multiple episodes of syncope. During the initial workup, he was noted to have sideroblastic anemia with hemoglobin of 4.5 grams per deciliter (g/dL), leukopenia, and elevated creatinine level suggesting acute kidney injury. Serum copper was 7 micrograms per deciliter (mcg/dL), and zinc was 2891 mcg/dL.

Abdominal radiographs showed several hundred coins in his upper gastrointestinal (GI) tract. During the initial hospitalization, he rapidly became pancytopenic. On hospital day 10, treatment was initiated with CaNa_2_EDTA and copper supplementation, followed by gastrotomy and removal of 212 coins. Many of the coins showed various degrees of dissolution and fragmentation. Approximately 50 coins could not be removed, and they were advanced into the colon during surgery. Zinc levels gradually declined, and the copper level increased. The anemia and renal failure resolved, and the patient was eventually discharged after 27 days of hospitalization.

Eight years later, the same patient presented to the ED with abdominal pain, vomiting, and pancytopenia. Plain radiographs once again showed numerous coins in the GI tract. Copper and zinc levels were noted to be < 3 mcg/dL and 1050 mcg/dL, respectively. On hospital day 2, he was treated with whole bowel irrigation (WBI) via nasogastric tube with one liter per hour of a polyethylene glycol-balanced salt solution (GoLYTELY) for one month (except for a five-day period when hospital stores were depleted). The pancytopenia was treated with intravenous (IV) copper sulfate and granulocyte colony-stimulating factor until his blood counts normalized. The patient defecated over 200 pennies during the course of his hospital stay. His zinc levels came down gradually, and his pancytopenia resolved. He was discharged home after 33 days of hospitalization.

Approximately 10 months later, routine outpatient blood work for this patient again showed pancytopenia, with low serum copper (< 3 mcg/dL) and elevated serum zinc levels (965 mcg/dL). Abdominal radiographs demonstrated multiple coins in the stomach, as well as a sharp-pointed screw. To avoid perforation he was taken to the operating room for foreign body removal. A total of 180 pennies and the screw were successfully retrieved with gastrotomy. IV copper was administered, the pancytopenia resolved, and the serum zinc concentration normalized. The patient was discharged home after 21 days hospitalization. Laboratory values from the three hospitalizations for this patient are provided in summary form in Table 1.

Data from all three hospitalizations were used to determine zinc pharmacokinetics. For half-life calculations, the initial zinc value was defined as the value most proximal to the initiation of treatment for zinc intoxication. The final zinc value was defined as that serum zinc level closest to one-fourth of the initial serum zinc concentration. For purposes of elimination kinetics, this was determined to represent two half-lives. Data from the half-life calculations for the three modalities are provided in Table 2.

## DISCUSSION

Copper deficiency caused by zinc intoxication with sideroblastic anemia and pancytopenia has been previous described.^8^,^12^–^15^ Because of complex interactions at absorptive sites in the small intestine, zinc toxicity can also lead to secondary copper deficiency. Copper and zinc are competitively absorbed in the proximal small intestine,^16^ and either can remain as a free metal or become bound to metallothionein (MT) and stored within enterocytes. Zinc bound to MT is excreted through the fecal route, within sloughed intestinal cells.^16^ The unbound zinc molecule, however, can be absorbed into the circulation and is unchanged with renal excretion.^16^ MT binds to copper with greater affinity than to zinc, and the MT-copper (Cu) complex is preferentially retained in the intestinal cells.^16^

**CPC-EM Capsule**

What do we already know about this clinical entity?

*Zinc poisoning is known to cause sideroblastic anemia. Chelation treatment has been suggested, but neither the benefit of chelation nor the best treatment are known*.

What makes this presentation of disease reportable?

*This is the first published report examining the effects of different treatment regimens for zinc poisoning in the same patient*.

What is the major learning point?

*Of the treatment options used, early surgical removal of the zinc had the greatest effect on the zinc levels. Copper supplementation also may be beneficial*.

How might this improve emergency medicine practice?

*Patients with zinc poisoning may present very ill. This case provides some guidance on early treatment of this unusual disease entity*.

The synthesis of MT is regulated by the amount of zinc ingested. When large amounts of zinc are ingested, more MT proteins are produced, forming more MT-Cu complexes, which are subsequently excreted.^16^ Massive zinc ingestion thereby decreases copper absorption, and leads to an increase in copper excretion.^14^,^16^ Since copper is a necessary cofactor for hematopoiesis,^17^ it is believed that copper deficiency prevents hematopoietic progenitor cells from replicating and differentiating, resulting in pancytopenia.^18^

Although zinc intoxication with pancytopenia due to massive ingestion of zinc-containing coins has been previously reported, the optimal treatment has not yet been established. The mortality risk associated with elemental zinc poisoning is also unknown, although a few previous case reports in humans have been associated with patient death.^3^,^19^ Based on a single case report^2^ chelation with CaNa_2_EDTA has also been suggested as a viable treatment. In that case, the patient was treated with surgery and eventually with chelation, but ultimately developed sepsis and died. It does not appear that the patient had ever received copper supplementation.^3^

Parenteral copper supplementation is another treatment option, as IV copper bypasses GI absorption. This may prevent copper binding with MT within the enterocytes and subsequent loss through intestinal cell sloughing. Based on the mechanism of zinc-induced copper deficiency, parenteral copper may improve the rate of immune reconstitution by supplementing copper stores for hematopoiesis. Our patient received IV copper supplementation with each hospitalization and normalized his white blood cell counts within one week of initiation of treatment, despite a significant zinc burden.

In our patient, it appears that the addition of CaNa_2_EDTA offered little benefit to enhance the elimination of zinc. The elimination rate associated with surgery alone is quite similar (80.0 vs 83.9 hours) to that for surgery plus CaNa_2_EDTA chelation. Theoretically, CaNa_2_EDTA chelation is believed to bind free zinc and prevent or reduce further MT expression, thereby reducing copper excretion. However, this kind of benefit could not be supported by these data.

## CONCLUSION

With the benefit of multiple presentations of the same patient with recurrent zinc toxicity, we were able to compare zinc elimination with whole bowel irrigation, surgery and surgery plus CaNa_2_EDTA chelation. Surgical removal of the zinc source (in these cases, copper-plated zinc U.S. pennies) seems to be the most important factor related to zinc elimination. Whole bowel irrigation alone resulted in a much longer elimination half-life. Chelation with CaNa_2_EDTA seemed to have little impact on the overall elimination of absorbed zinc. As parenteral exogenous copper was provided during each hospital admission, the effects of copper supplementation cannot be quantified based on these data. In the one previously reported fatality from massive coin ingestion, copper was not supplemented.^3^

Based upon the mechanism of zinc-induced copper deficiency, parenteral copper supplementation may be of benefit in restoring hematopoeisis, by bypassing the GI tract. However, there is currently minimal data to support or refute its use in treating this disease entity. At this time, the optimal treatment for zinc intoxication resulting in copper deficiency from massive ingestion remains unknown. Based on our experience, however, surgical decontamination with parenteral copper supplementation should be considered. Furthermore, based upon this limited case series with the same patient, decontamination with whole bowel irrigation may be inferior to surgery, and chelation with CaNa_2_EDTA may offer no improvement in elimination half-life.

## Figures and Tables

**Table 1:** Serum laboratory values on each of the three visits for zinc toxicity.

Admission number	Treatment	Time (h)	Initial Zn (mcg/dL)	Final Zn (mcg/dL)	Elimination half-life (h)
1	Chelation + Surgery	163.0	1720	419	80.0
2	WBI	467.0	1050	262	233.2**
3	WBI + Surgery	147.3	965	286	83.9

** There are two conflicting samples for this calculation, and we suspect that there was an incorrect time stamp on one of them. If we use the alternative sample, which was drawn after initiation of whole bowel irrigation, this value drops to 162 hours.

*h*, hours; *Zn*, zinc; *mcg/dL*, micrograms per deciliter; *WBI*, whole bowel irrigation.

**Table 2:** Elimination kinetics for the three modalities of treatment.

Laboratory value	Visit 1	Visit 2	Visit 3	Normal range
Hemoglobin (g/dL)	4.5	4.6	4.0	13.3 – 17.1
Creatinine (mg/dL)	1.5	n/a	n/a	0.6 – 1.1
WBC (K/mm^3^)	2.5	1.1	1.4	3.5 – 10.6
Platelet count (K/mm^3^)	125	89	149	150 – 460
Copper (mcg/dL)	7	<3	<3	70 – 155
Zinc (mcg/dL)	2891	1050	965	60 – 130

*g/dL*, grams per deciliter; *mg/dL*, micrograms per deciliter; *WBC*, white blood cells; *K/mm^3^*, thousand cells per cubic millimeter; *mcg/dL*, micrograms per deciliter; *n/a*, not available.
